# Non-local classical optical correlation and implementing analogy of quantum teleportation

**DOI:** 10.1038/srep09175

**Published:** 2015-03-17

**Authors:** Yifan Sun, Xinbing Song, Hongwei Qin, Xiong Zhang, Zhenwei Yang, Xiangdong Zhang

**Affiliations:** 1 School of Physics, Beijing Institute of Technology, 100081, Beijing, China; 2 Department of Physics, Beijing Normal University, Beijing 100875, China

## Abstract

This study reports an experimental realization of non-local classical optical correlation from the Bell's measurement used in tests of quantum non-locality. Based on such a classical Einstein–Podolsky–Rosen optical correlation, a classical analogy has been implemented to the true meaning of quantum teleportation. In the experimental teleportation protocol, the initial teleported information can be unknown to anyone and the information transfer can happen over arbitrary distances. The obtained results give novel insight into quantum physics and may open a new field of applications in quantum information.

Quantum teleportation provides a means to transport an unknown quantum state from one location to another over arbitrary distances. Since originally being proposed in 1993^1^, the study on quantum teleportation has aroused great interest. Its experimental realizations in various different ways have been reported. For instance, the best known methods have been implemented using photon polarization and optical techniques^2^,^3^, by means of squeezed states of light^4^, by applying nuclear magnetic resonance (NMR) techniques^5^, and by a hybrid technique^6^,^7^. Teleportation has also been accomplished between photons and a single atomic ensemble^8^,^9^, between distant atomic objects^10^,^11^,^12^,^13^, and in a chip-based superconducting circuit architecture^14^. Quantum teleportation is essential for large-scale quantum communication^15^,^16^,^17^ and distributed quantum networks^18^. It has proven to be a useful tool for realizing universal quantum logic gates in quantum computing and general quantum information manipulation^19^. However, all protocols for accomplishing quantum teleportation require non-local correlations, or Einstein–Podolsky–Rosen (EPR) entanglement, between systems shared by the sender and receiver.

On the other hand, non-separable correlations among two or more different degrees of freedom from the same classical optical beam, have been discussed^20^,^21^,^22^,^23^,^24^,^25^,^26^,^27^,^28^,^29^,^30^,^31^,^32^. The violation of Bell's inequality or GHZ theorem for such a non-separable correlation has been demonstrated experimentally^20^,^21^,^22^,^23^,^24^,^25^. Thus, a non-separable classical correlation is called “nonquantum entanglement” or “classical entanglement”^25^,^26^,^27^,^28^,^29^,^30^. Such a classical entanglement has been applied to resolve basic issues in polarization optics^25^, simulate quantum walks, et al.^27^. The investigations have shown that quantum optical procedures requiring entanglement without non-locality can actually be achieved in classical optics regime^25^,^26^,^27^,^28^,^29^,^30^,^31^,^32^. Recently, some discussions on the non-local classical optical correlation of two coherent light beams have been done^33^,^34^,^35^,^36^. Some studies, which have attempted to simulate the quantum teleportation by classical optics, have been performed^29^,^30^,^31^. However, the classical correspondence on the true meaning of quantum teleportation, which is based on the classical EPR correlation state, has not been achieved. In this work, we present a new method to construct a non-local classical EPR correlation state by using two incoherent light beams and implement a classical analogy on the true meaning of quantum teleportation.

## Results and Discussion

### Non-local classical optical correlation

**Figure 1:**
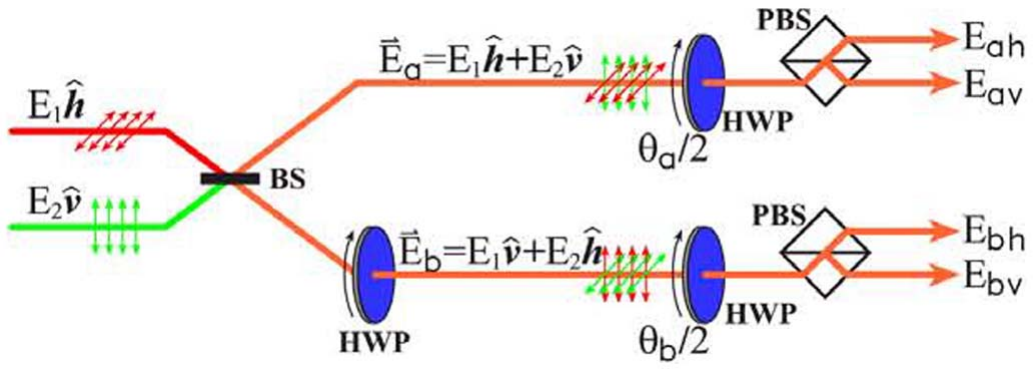
Experimental setup for CHSH-type Bell inequality violation by using two separable classical light sources. E_1_ and E_2_ are two laser beams with different wavelengths. 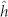 and 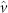 denote the horizontal and vertical polarization components. (P)BS, (polarizing) beam splitter; HWP, half-wave plate. The first-order correlation measurement is performed between *E_ai_* and *E_bj_* (*i*, *j* = *h*, *v*).

Initially two independent light beams, *E*_1_ and *E*_2_ with different wavelengths, are considered and pass through a 50/50 beam splitter (BS) as shown in Fig. 1. The fields of two light beams satisfy complete incoherent condition 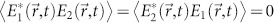 and 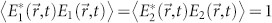. Here 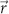 and *t* represent coordinates of space and time. After the BS and a half-wave plate (HWP) in one output of the BS, two new outputs with 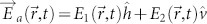 and 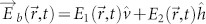 can be obtained, where 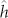 and 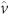 refer to the horizontal and vertical polarization components, respectively. In order to analyze the correlation properties between 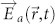 and 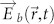, a half-wave plate (HWP) and a polarizing beam splitter (PBS) are introduced in each path. They function to rotate angles of the light beam and separate 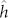 and 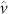 polarized light. Here *θ_a_* and *θ_b_* represent polarization rotated angles in two paths, respectively. After the HWP and PBS, two polarized beams become four beams. Their fields are described by *E_ah_*, *E_av_*, *E_bh_* and *E_bv_*, respectively, which can be modulated by *θ_a_* and *θ_b_*.

Now we perform the famous Clauser–Horne–Shimony–Holt (CHSH) test in our system as shown in Fig. 1. The correlation function is defined in the following form^20^:
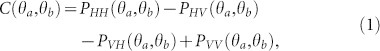
where *P_HH_* = *N_HH_* (*θ_a_*,*θ_b_*)/*N_Total_*, *P_VV_* = *N_VV_* (*θ_a_*,*θ_b_*)/*N_Total_*, *P_HV_* = *N_HV_* (*θ_a_*,*θ_b_*)/*N_Total_*, and *P_VH_* = *N_VH_* (*θ_a_*,*θ_b_*)/*N_Total_* are normalized correlated probabilities when polarizations of each beam are measured, and *N_Total_* = *N_HH_* (*θ_a_*,*θ_b_*) + *N_VV_* (*θ_a_*,*θ_b_*) + *N_HV_* (*θ_a_*,*θ_b_*) + *N_VH_* (*θ_a_*,*θ_b_*). In our experiment, such correlated probabilities can be represented by the first order correlation of electric fields through *N_IJ_* (*θ_a_*,*θ_b_*) (I(J) represents H or V):
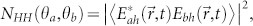

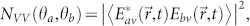

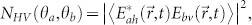

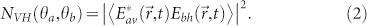
In the experiment the fields of output lights are not directly measured. However, the first-order field correlation can be obtained through measuring the difference of light intensities at two export positions on Mach-Zehnder interferometer, because 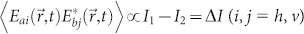. Here *I*_1_ and *I*_2_ represent the light intensities at two export positions of the Mach-Zehnder interferometer and Δ*I* is the difference between them. More information about the measurement method for the first-order field correlation is provided in the Methods section.

**Figure 2:**
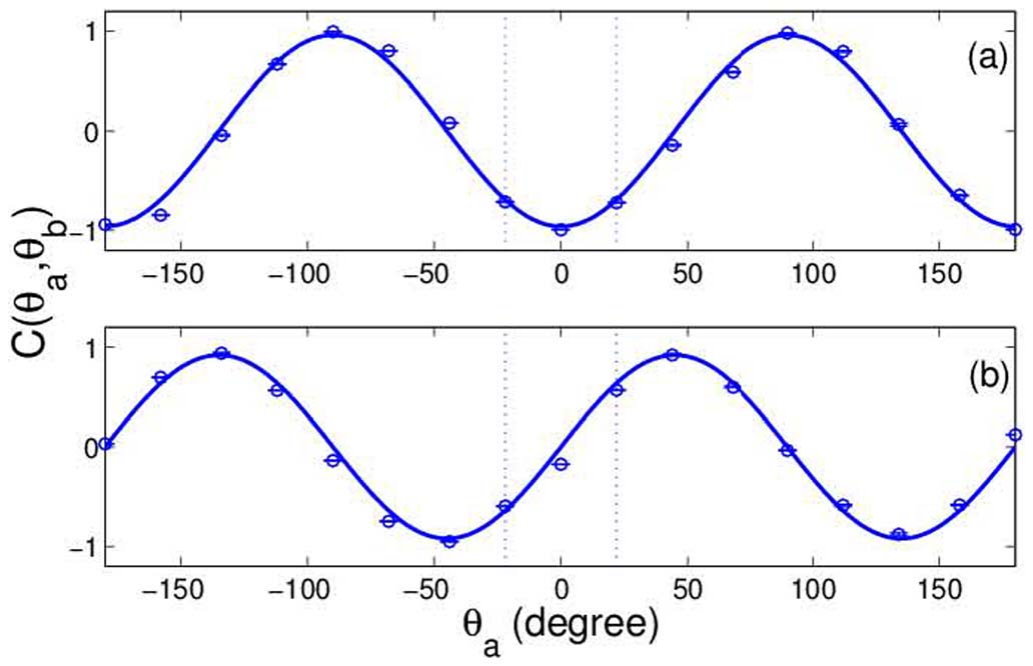
The correlation functions *C*(*θ_a_*,*θ_b_*) as a function of polarization rotated angle *θ_a_* at *θ_b_* = 0° (a) and *θ_b_* = 45° (b). The circle dots and solid lines represent the experimental and theoretical results, respectively. The dashed lines mark the values of *θ_a_* to achieve the maximum violations of Bell inequalities.

Figure 2 shows experimental results for the correlation functions *C*(*θ_a_*,*θ_b_*) as a function of polarization rotated angle *θ_a_* at some certain *θ_b_*. Here two laser beams (continuous wave mode) with different wavelengths, 532 *nm* and 632.8 *nm*, are used. Their intensities are taken as 2 mW. The circle dots and solid lines represent the experimental measurements and theoretical results, respectively. It can be seen that the experimental results are in good agreement with the theoretical calculations. After we have obtained *C*(*θ_a_*,*θ_b_*), the CHSH formulation of Bell's measurement is used to quantify such correlation. The CHSH measurement is
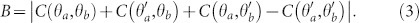
It is well known that the local realism theories give |*B*| < 2. However, from the experimental results marked by dashed lines in Fig. 2, *B* = 2.597 ± 0.012 > 2 is obtained as *θ_a_* = *π*/4, *θ_b_* = 0, 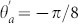 and 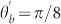. The presented experiment yields the strongest violation of Bell's inequalities.

Any of the four EPR-Bell states can be constructed by adjusting polarization and phase factor via HWP in the above experimental setup. This is highly similar to the production of polarization-entangled photon pairs from spontaneous down-conversion of nonlinear crystals^37^. For example, the output fields 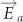 and 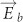 shown in Fig. 1 correspond to the following symmetric Bell-state:
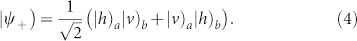
Here we use the notation |*h*)*_i_*(|*v*)*_i_*) to express a classical state^29^,^31^. That is to say, if 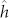 polarization is measured in one beam, the information of 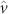 polarization can be “determined” in another beam due to the first-order field correlation, and vice versa. This means that a classical correlation state has been constructed. Because the correlation between two beams is independent on the separation between them, such a correlation is non-local and can be regarded as a classical analogy of EPR entangled state in quantum mechanics. The problem is whether or not some unique phenomena such as quantum teleportation can be realized by applying such a classical EPR correlation state, which is similar to the case in quantum information process.

### Classical analogy of quantum teleportation based on non-local classical correlation

**Figure 3:**
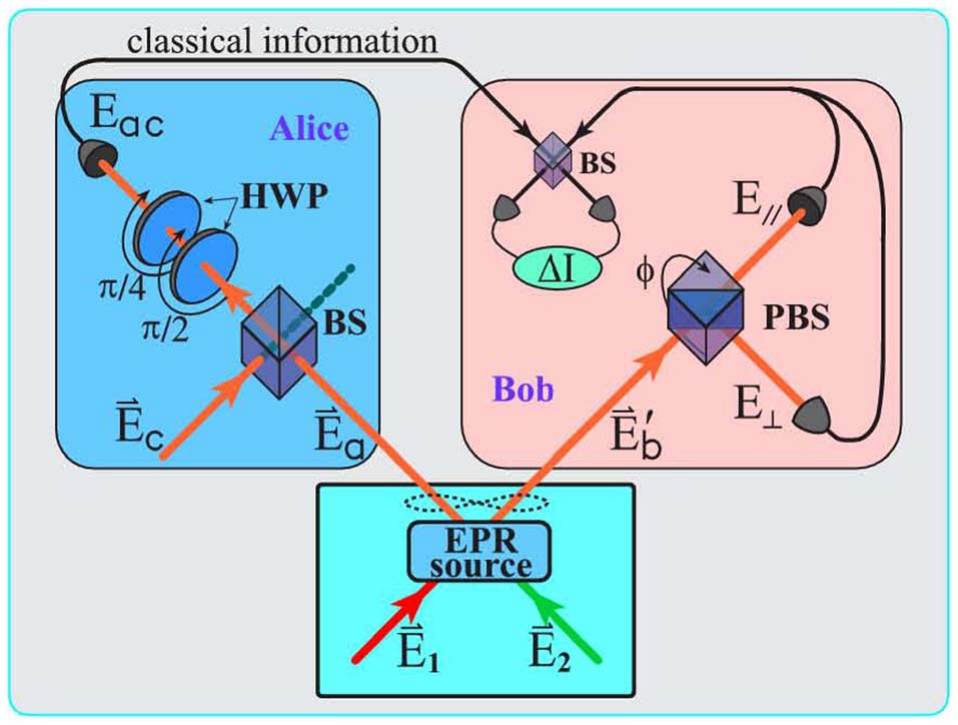
Teleportation scheme showing principles and experimental set-up for the linear polarization case. The classical EPR source shown in the bottom plate is the same to that in Fig. 1. Alice and Bob share an ancillary classical entangled states marked by 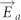 and 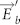. Alice performs a joint Bell-state measurement on 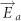 and the initial state marked by 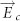. After Alice has sent the measured result *E_ac_* as classical information to Bob, Bob performs the correlation measurement of the first-order field by using 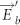 and *E_ac_*. *E*_||_ and 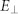 represent the transmission and reflection parts as 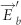 passes through a rotated PBS, Δ*I* is the difference of the light intensities at two export positions.

In order to study the classical analogy of quantum teleportation, the experimental setup shown in Fig. 3 is considered. The experimental generation of EPR correlation states and Bell-state measurement are two key parts in the teleportation scheme. In this scheme, above classical EPR correlation states are used as the source. A three-photon scheme is referred to for the Bell-state measurement as described by D. Bouwmeester et. al.^2^. This system features the antisymmetric state obtained from four Bell-states. Thus, the antisymmetric Bell-state is used in our teleportation scheme: 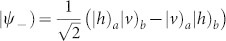. This corresponds to 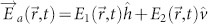 and 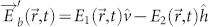 in our classical EPR system, which are shared by Alice and Bob, for example. If Alice want to teleport an initial state |*ψ_c_*) = *c*_1_ |*h*) + *c*_2_ |*v*) to Bob, she needs perform a joint Bell-state measurement on the initial state and 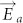. In the experiments, the field corresponding to the initial state |*ψ_c_*) is expressed as: 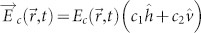, where 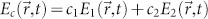, *c*_1_ and *c*_2_ satisfy |*c*_1_|^2^ + |*c*_2_|^2^ = 1. When *c*_1_ and *c*_2_ are considered real, corresponds to the linear polarization case. Whereas, when *c*_1_ and *c*_2_ are complex, it is in correspondence to the circular polarization case. We first consider linear polarization case, in which *c*_1_ and *c*_2_ can be expressed by the direction angle of polarization *θ*: *c*_1_ = cos *θ* and *c*_2_ = sin *θ*. Thus, *θ* will be the teleported information.

In order to perform a joint Bell-state measurement on the initial state and 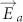, an optical element group is constructed that includes a BS and two HWPs as shown in Fig. 3. There are two output ports of the BS, in which one is the sum of two input fields, and the other is the difference of the two. Two HWPs are placed at the output port of the difference. The functions of two HWPs are to realize interchange between 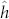 and 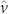 polarizations, and add a *π* phase for the component of 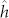 polarization field. For such an optical element group, if the input ports are in the Bell-states, it is easy to demonstrate by the correlation measurement of the first-order field that only the output of the antisymmetric state is not zero, and the outputs of three symmetric states all are zero (see Methods section). This is similar to the scheme of the Bell measurements in Ref. 2. Referring back to the previous example, and considering synchronicity of Alice's and Bob' measurements, Alice obtains the information *E_ac_* as 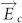 and 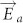 enter the optical element group, and then sends it to Bob through the classical channel. Here *E_ac_* represents the amplitude of field without any polarization information (see Supplementary). Owing to lack of technique to measure fields, we had to send the beam to Bob in the experiment. Next, we show that Bob can only use the coherence property of the field rather than the polarization information, and such a process is corresponded to the method used in Ref. 2.

After Bob receives the information that Alice sent, he will perform the correlation measurement of the first-order field by using the information and 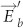 in order to obtain the teleported material. This is because there is a strong correlation between *E_ac_* and 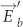 (see Supplementary). For example, as 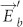 passes through a rotated PBS, it will be orthogonally decomposed into *E*_//_ and 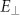 as shown in Fig. 3. Our theoretical calculations show that |〈*E_ac_E*_//_〉|^2^ = cos^2^ (*θ* − *ϕ*) and 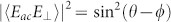, which are the correlation of field without polarization (see Supplementary). Here *ϕ* represents the rotated angle of the PBS, and is taken as 0° when the polarization of transmission fields is horizontal. From these relations, it is not difficult to find that *θ* and *ϕ* are in one-to-one correspondence. If Bob has obtained the maximum of the first-order correlation between *E_ac_* and *E_//_* for a polarization direction, the corresponding *ϕ* for such a polarization direction is the teleported information *θ* (see Supplementary).

**Figure 4:**
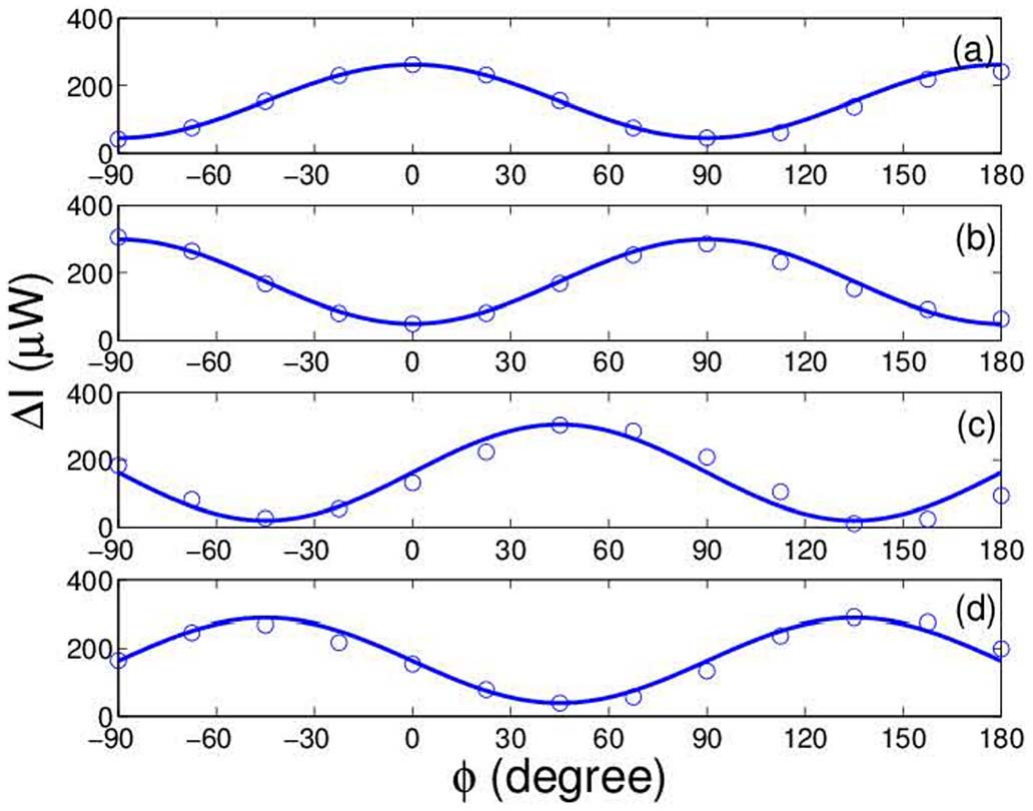
Experimental (circle dots) and theoretical (solid lines) results from the correlation measurement of the first-order field for the linear polarization case, which are described by the differences of light intensities Δ*I* as a function of the angle *ϕ*. (a) and (b) correspond to the results for *E*_||_ and 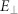, respectively, as the polarization of the initial state *θ* = 0; (c) and (d) display the corresponding results as *θ* = *π*/4.

In the experiment, the first-order correlations between *E_ac_* and *E_//_* (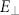) are measured by the difference of light intensities, which is similar to the above method for testing CHSH formulation. Figure 4 displays the experimental results for the first-order correlation degree, Δ*I* = *I*_1_ − *I*_2_, as a function of the angle *ϕ*. Figure 4(a) and (b) correspond to the case with *θ* = 0 for *E*_//_ and 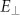, respectively, whereas the corresponding results for *θ* = *π*/4 are plotted in Fig. 4(c) and (d). From Fig. 4(a), the maximum of the first-order correlation appears at *ϕ* = 0, which is in agreement with *θ* = 0. At the same time, the minimum of the first-order correlation is also found at an orthogonal direction as shown in Fig. 4(b), which validates realization of the teleportation process. Similarly, as *θ* = *π*/4, Bob has measured the maximum of the first-order correlation at *ϕ* = *π*/4 and the minimum also appears at its orthogonal direction (Fig. 4(c) and (d)). In fact, the above design is suitable for any linear polarization state. The corresponding experiment to teleport a circular polarized initial state was also performed by using the above scheme, similar teleportation process has been realized (see Supplementary). In the teleportation process, it is not necessary for Alice to know where Bob is, the initial polarization state can be unknown to anyone not only Alice. Furthermore, the transfer of information from Alice to Bob can happen over arbitrary distances. These properties for the teleportation in the presented scheme completely agree with those described by D. Bouwmeester et. al., which this work' results focus on^2^. Discovery of other schemes for quantum teleportation by using classical optics warrants further study.

## Conclusions

We have demonstrated experimentally the non-local classical optical correlation from the Bell's measurement used in tests of quantum non-locality. Based on such a classical EPR correlation, a classical analogy has been implemented to the true meaning of quantum teleportation. The presented results indicate that some non-local phenomena in quantum machines can be realized in classical optical signal processing. Thus, this study opens a new way to obtain the quantum information process in the classical optical communication network. It not only provokes deep thought on some basic physical problems such as essence of entanglement and correlation, but also shows potential application in classical and quantum information processes.

## Methods

### Measurement method for the first-order field correlation

**Figure 5:**
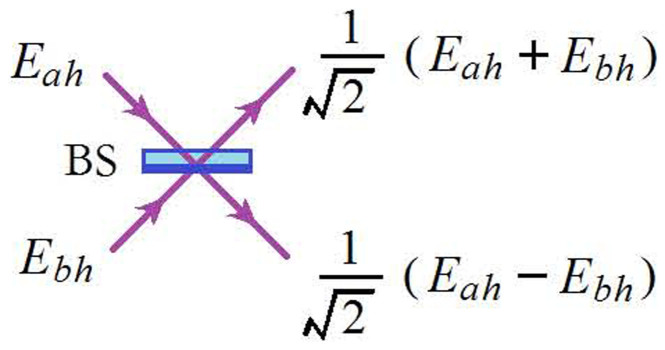
Schematic picture for the first-order field correlation.

In general, the polarization-entangled photon pairs can be produced from spontaneous down-conversion of the nonlinear crystal. In such a process, the photon states for 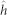 and 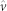 polarizations are generated with a certain probability at the same time, which the entangled properties can be measured by the coincidence counts. Based on the coherence of light field, we can construct the corresponding non-local classical EPR correlation states as described by Eq.(4), which the correlated properties can be shown by the first-order field correlation. Basically, the measure of the first-order field correlation can be realized by synchronous local measurements and doesn't require direct contact. However, in the experiment it is difficult to obtain the first-order field correlation by directly measuring fields. In addition, the synchronization is very hard to achieve. So, we take the following method, that is, take 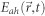 and 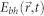 for example and do the following Hadamard transformation:
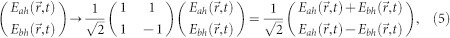
then
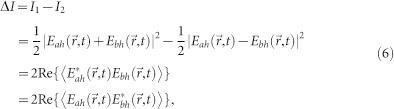
where Re{X} represents the real part of X. From the experimental setup in Fig.1, we can find 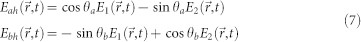
From complete incoherent condition 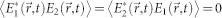 and 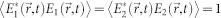,it is easy to find the following relation:
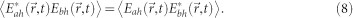
Then
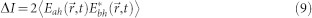
That is, the first-order field correlation can be measured by the difference of light intensities and this method is also effective in other measurements of experiments. The above process can be realized by the beam splitter as shown in Figure 5. In the experiment, the same polarization for the light beams is required.

### Realization of Bell-state measurement for classical optical entangled states

**Figure 6:**
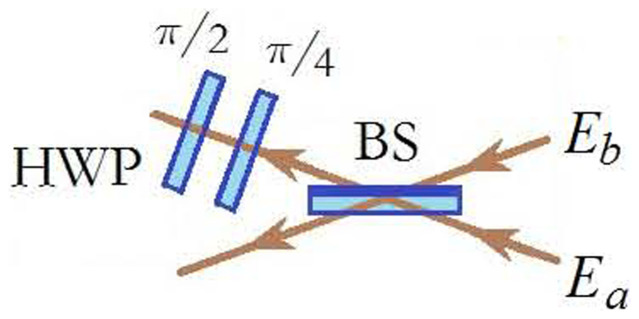
Design scheme for Bell-state measurement.

Bell-state measurement is a key part in the teleportation scheme. In our teleportation scheme, we perform the Bell-state measurement referring to three-photon scheme described in Ref. 2 as shown in Figure 6. The marks *π*/2 and *π*/4 in Figure 6 represent the angle between the axis of HWP and the horizontal direction. The functions of two HWPs are to realize interchange between 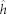 and 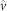 polarizations, add a *π* phase for the component of 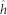 polarization field. The Jones matrix for the BS is taken in the following form:
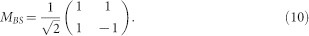
Two HWPs are put at the output port of the difference. If the input ports are in four Bell-states, the results by the correlation measurement of the first-order field are given in the table 1.

Taking the antisymmetric state as an example, in the following we give a demonstration on such a result. The antisymmetric Bell state is
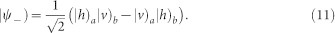
The corresponding field is
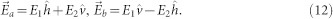
After passing through the BS, they become
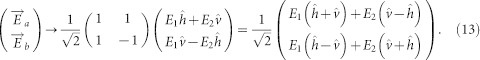
Consider the output of the difference, perform interchange between 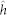 and 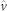 polarizations and add a *π* phase for the component of 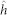 polarization field by the HWPs, we have
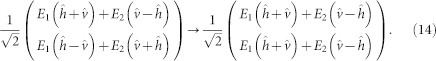
From the complete incoherent condition and the correlation measurement of the first-order field for two components in Eq.(14), we have:
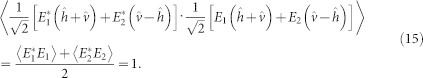
Similarly, consider three symmetric states passing through the above optical element group, we find that all outputs are zero. Here the effect of polarization is considered in the correlation measurement of the first-order field.

## Author Contributions

Theoretical method is presented by Y.S., the corresponding experiments are performed by X.S. Thus, Y.S. and X.S. contributed equally to this work. In doing the experiments, X.S. get the help of H.Q., X.Z. and Z.Y., the idea and physical analysis are given by X.Z. All authors reviewed the manuscript.

## Supplementary Material

Supplementary Information Supplementary Information

## Figures and Tables

**Table 1:** The results for Bell-state measurement

Bell-state	Classical field E_a_	Classical field E_b_	The first-order field correlation
|*φ*_+_)	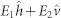	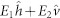	0
|*φ*_−_)		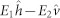	0
|*ψ*_+_)		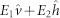	0
|*ψ*_−_)		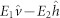	1
