# Supplementary material for: Non-local classical optical correlation and implementing analogy of quantum teleportation
Source: Sci Rep. 2015 Mar 17;5:9175. doi: 10.1038/srep09175 (PMC4361852; doi:10.1038/srep09175)
Supplement: Supplementary Information [file srep09175-s1.pdf]

Supplementary Information

Non-local classical optical correlation and implementing analogy of quantum teleportation

Yifan Sun,[1,2] Xinbing Song[1], Hongwei Qin,[1] Xiong Zhang,[1] Zhenwei Yang,[1] and Xiangdong Zhang[1]

[1]School of Physics, Beijing Institute of Technology, 100081, Beijing, China, [2]Department of Physics, Beijing Normal University, Beijing 100875, China.

The basic principle of quantum teleportation for our design. In the following, we introduce the implementation process of our teleportation scheme in detail. As introduced in the text, the fields being taken by us are： $\vec{E}_a = E_1\hat{h} + E_2\hat{v}$ and $\vec{E}_b = E_1\hat{v} - E_2\hat{h}$. The corresponding Bell state is given in Eq.(11). If we want to teleport an information state $\left|\psi_C\right\rangle = c_1\left|h\right\rangle_c + c_2\left|v\right\rangle_c$, we need construct the following state:

$$
\begin{aligned}
\left|\psi_{Total}\right\rangle &= \left|\psi_C\right\rangle \otimes \left|\psi_-\right\rangle \\
&= \frac{1}{\sqrt{2}}\left(c_1\left|h\right\rangle_c + c_2\left|v\right\rangle_c\right)\otimes\left(\left|h\right\rangle_a\left|v\right\rangle_b - \left|v\right\rangle_a\left|h\right\rangle_b\right) \\
&= \frac{c_1}{\sqrt{2}}\left(\left|h\right\rangle_c\left|h\right\rangle_a\left|v\right\rangle_b - \left|h\right\rangle_c\left|v\right\rangle_a\left|v\right\rangle_b\right) \\
&\quad + \frac{c_2}{\sqrt{2}}\left(\left|v\right\rangle_c\left|h\right\rangle_a\left|v\right\rangle_b - \left|v\right\rangle_c\left|v\right\rangle_a\left|h\right\rangle_b\right)
\end{aligned} \qquad \text{(S1)}
$$

Defining the Bell state between $\vec{E}_a$ and $\vec{E}_c$:

$$
\begin{aligned}
\left|\psi_{ca}^{(\pm)}\right\rangle &= \frac{1}{\sqrt{2}}\left(\left|h\right\rangle_c\left|v\right\rangle_a \pm \left|v\right\rangle_c\left|h\right\rangle_a\right) \\
\left|\varphi_{ca}^{(\pm)}\right\rangle &= \frac{1}{\sqrt{2}}\left(\left|h\right\rangle_c\left|h\right\rangle_a \pm \left|v\right\rangle_c\left|v\right\rangle_a\right)
\end{aligned} \qquad \text{(S2)}
$$

To express $\left|\psi_{Total}\right\rangle$ by Eq.(S2), we obtain

$$
\begin{aligned}
\left|\psi_{Total}\right\rangle = \frac{1}{2}\Big[&\left|\varphi_{ca}^{(+)}\right\rangle\left(c_1\left|v\right\rangle_b - c_2\left|h\right\rangle_b\right) + \left|\varphi_{ca}^{(-)}\right\rangle\left(c_1\left|v\right\rangle_b + c_2\left|h\right\rangle_b\right) \\
&- \left|\psi_{ca}^{(+)}\right\rangle\left(c_1\left|h\right\rangle_b - c_2\left|v\right\rangle_b\right) - \left|\psi_{ca}^{(-)}\right\rangle\left(c_1\left|h\right\rangle_b + c_2\left|v\right\rangle_b\right)\Big]
\end{aligned} \qquad \text{(S3)}
$$

From Eq.(S3), we find that there is a strong correlation between the antisymmetric state $\left|\psi_{ca}^{(-)}\right\rangle$ and the polarization state $c_1\left|h\right\rangle_b + c_2\left|v\right\rangle_b$ for Bob. Such a polarization state for Bob is identical with that of the initial polarization state in Alice side. Such a result is agreement with the theoretical description in Ref.[1]. In fact, such a process can also be realized by the description of fields. The field corresponding to the initial state $\left|\psi_c\right\rangle$ is given in the text: $\vec{E}_c = E_c\left(c_1\hat{h} + c_2\hat{v}\right)$, where $E_c\left(\vec{r},t\right) = c_1E_1\left(\vec{r},t\right) + c_2E_2\left(\vec{r},t\right)$, $c_1 = \cos\theta$ and $c_2 = \sin\theta$ for the linear polarization case. For purposes of analysis, we do decomposition for $\vec{E}_a$ and $\vec{E}_b$, and introduce the following base vector transformation:

$$\begin{aligned} &\hat{a}_{//} = \cos\theta\hat{h} + \sin\theta\hat{v}, \hat{a}_{\perp} = -\sin\theta\hat{h} + \cos\theta\hat{v} \\ &\hat{b}_{//} = \cos\phi\hat{h} + \sin\phi\hat{v}, \hat{b}_{\perp} = -\sin\phi\hat{h} + \cos\phi\hat{v} \end{aligned}, \quad \text{(S4)}$$

where $\theta = \arctan\left(c_2 / c_1\right)$ and φ can be any value. Then

$$\begin{aligned} \vec{E}_a &= E_1\hat{h} + E_2\hat{v} \\ &= \left(\vec{E}_a \cdot \hat{a}_{//}\right)\hat{a}_{//} + \left(\vec{E}_a \cdot \hat{a}_{\perp}\right)\hat{a}_{\perp} \\ &= \left(\cos\theta E_1 + \sin\theta E_2\right)\hat{a}_{//} \\ &+ \left(-\sin\theta E_1 + \cos\theta E_2\right)\hat{a}_{\perp} \end{aligned} \quad \text{(S5)}$$

and

$$\begin{aligned} \vec{E}'_b &= E_1\hat{v} - E_2\hat{h} \\ &= \left(\vec{E}_b \cdot \hat{b}_{//}\right)\hat{b}_{//} + \left(\vec{E}_b \cdot \hat{b}_{\perp}\right)\hat{b}_{\perp} \\ &= \left(\sin\phi E_1 - \cos\phi E_2\right)\hat{b}_{//} \\ &+ \left(\cos\phi E_1 + \sin\phi E_2\right)\hat{b}_{\perp} \end{aligned}. \quad \text{(S6)}$$

The above decompositions are valid for arbitrary θ and φ. Then, Alice performs Bell measurements for $\vec{E}_a$ and $\vec{E}_c$ by using the BS. The process of measuring experience is as follows:

$$\begin{aligned} &\vec{E}_a, \vec{E}_c \\ &\xrightarrow{BS} \frac{1}{\sqrt{2}}\left(\vec{E}_a - \vec{E}_c\right) = \frac{1}{\sqrt{2}}\left(-\sin\theta E_1 + \cos\theta E_2\right)\left(-\sin\theta\hat{h} + \cos\theta\hat{v}\right) \end{aligned}$$

$\downarrow$ $\pi/4$ HWP

$$\frac{1}{\sqrt{2}}\left(-\sin\theta E_1 + \cos\theta E_2\right)\left(-\sin\theta\hat{v} + \cos\theta\hat{h}\right)$$

$\downarrow$ $\pi/2$ HWP

$$\vec{E}_{ac} = -\left(-\sin\theta E_1 + \cos\theta E_2\right)\left(\cos\theta\hat{h} + \sin\theta\hat{v}\right). \tag{S7}$$

Finally we need only the field part $E_{ac} = -\sin\theta E_1 + \cos\theta E_2$ of $\vec{E}_{ac}$ as the measurement result to be sent to Bob. Such a result is obtained by the optical element group as shown in Fig.3. In the quantum teleportation scheme described in Ref.[2], Bell measurement is performed by the BS, two photon HOM interference is also realized by BS, the difference between two photon is recorded by the coincidence counts. Our present scheme has the same effect with that in Ref.[2]. On the Bob side, orthogonal decomposition, or polarization detection, can be realized by rotating the PBS. It is expressed as

$$\begin{aligned} \vec{E}_{//} &= \left(\sin\phi E_1 - \cos\phi E_2\right)\hat{b}_{//} \\ \vec{E}_{\perp} &= \left(\cos\phi E_1 + \sin\phi E_2\right)\hat{b}_{\perp} \end{aligned}. \tag{S8}$$

From Eq.(S3), we are sure that the correlation intensity for the measured results $\left|\psi_{ca}^{(-)}\right\rangle$ and $c_1\left|h\right\rangle_b + c_2\left|v\right\rangle_b$ appears the maximum. In fact, E_{ac} is the measured result for the $\left|\psi_{ca}^{(-)}\right\rangle$. Thus, the angle ϕ, which is determined by the maximum of the first-order correlation between E_{ac} and $E_{\square}$, corresponds to the teleported information θ. The first-order correlation between $E_{\perp}$ and orthogonal components of the field appears the minimum. From the above relationships, we can obtain

$$\left|\left\langle E_{ac}E_{//}\right\rangle\right|^2 = \cos^2\left(\theta-\phi\right)$$

$$\left|\left\langle E_{ac}E_{\perp}\right\rangle\right|^2 = \sin^2\left(\theta-\phi\right). \tag{S9}$$

The experiment results of teleportation for the circular polarization light. In order to realize the teleportation for the initial state with the circular polarization case, the EPR states need be written as a circular polarization form. From the following base vector transformation,

$$\begin{aligned}\hat{h} &= \left(\hat{r}+\hat{l}\right)/\sqrt{2} \\ \hat{v} &= \left(\hat{r}-\hat{l}\right)/\left(i\sqrt{2}\right)\end{aligned}, \tag{S10}$$

the fields $\vec{E}_a$ and $\vec{E}_b'$ in the main text can be rewritten as

$$\begin{aligned}\vec{E}_a &= E_1\left(\vec{r},t\right)\hat{h}+E_2\left(\vec{r},t\right)\hat{v} \\ &=\left[\left(E_1-iE_2\right)\hat{r}+\left(E_1+iE_2\right)\hat{l}\right]/\sqrt{2}\end{aligned} \tag{S11}$$

and

$$\begin{aligned}\vec{E}_b' &= E_1\left(\vec{r},t\right)\hat{v}-E_2\left(\vec{r},t\right)\hat{h} \\ &=\left[\left(E_1-iE_2\right)\hat{r}-\left(E_1+iE_2\right)\hat{l}\right]/\left(i\sqrt{2}\right)\end{aligned}. \tag{S12}$$

Consider noncoherent hypothesis for E_1 and E_2, the first-order correlation between E_1+iE_2 and E_1-iE_2 is similar to orthogonality relations of base vectors for the circular polarization case:

$$\begin{aligned}\left\langle \frac{\left(E_1-iE_2\right)}{\sqrt{2}}\frac{\left(E_1-iE_2\right)^*}{\sqrt{2}}\right\rangle &= \left\langle \frac{\left(E_1+iE_2\right)}{\sqrt{2}}\frac{\left(E_1+iE_2\right)^*}{\sqrt{2}}\right\rangle = 1 \\ \left\langle \frac{\left(E_1+iE_2\right)}{\sqrt{2}}\frac{\left(E_1-iE_2\right)^*}{\sqrt{2}}\right\rangle &= \left\langle \frac{\left(E_1-iE_2\right)}{\sqrt{2}}\frac{\left(E_1+iE_2\right)^*}{\sqrt{2}}\right\rangle = 0\end{aligned}. \tag{S13}$$

If we let

$$\begin{aligned}E_l &= \left(E_1+iE_2\right)\big/\sqrt{2} \\ E_r &= \left(E_1-iE_2\right)\big/\sqrt{2}\end{aligned}, \tag{S14}$$

the light fields in two paths can be rewritten as:

$$\begin{aligned}\vec{E}_a &= E_r\hat{r} + E_l\hat{l} \\ \vec{E}'_b &= -i\left(E_r\hat{r} - E_l\hat{l}\right)\end{aligned}. \tag{S15}$$

The corresponding EPR entanglement state can be written as：

$$|\psi'\rangle = \frac{1}{\sqrt{2}}\left(|r\rangle_a|r\rangle_b - |l\rangle_a|l\rangle_b\right). \tag{S16}$$

The scheme's main aim for Bell measurement is that the antisymmetric state can be obtained from four Bell-states. Thus, we rewrite the state described by Eq.(S16) in the form of the antisymmetric state by using HWP:

$$|\psi_-\rangle = \frac{1}{\sqrt{2}}\left(|l\rangle_a|r\rangle_b - |r\rangle_a|l\rangle_b\right). \tag{S17}$$

The corresponding fields are：

$$\begin{aligned}\vec{E}_a &= E_r\hat{r} + E_l\hat{l} \\ \vec{E}''_b &= -i\left(E_r\hat{l} - E_l\hat{r}\right)\end{aligned}. \tag{S18}$$

The expressions of Eq.(S18) are identical with those of the linear polarization case. Thus, theory and scheme of teleportation for the circular polarization case is also similar to the case of the linear polarization. The experimental set-up for the teleportation of the circular polarization initial state is given in Figure D1. Let Alice teleport an initial state $|\psi_c\rangle = c_1|h\rangle + c_2|v\rangle$ to Bob, here $c_1 = 1/\sqrt{2}$ and $c_2 = i/\sqrt{2}$ for the circular polarization case, the corresponding field is expressed as： $\vec{E}'_c = E_r\hat{r} = \frac{1}{2}(E_1 - iE_2)\left(\hat{h} + i\hat{v}\right)$.

The method to perform a joint Bell-state measurement on the initial state for the circular polarization case is different from that of the linear polarization case. In the teleportation scheme for the circular polarization case, only one HWP is needed. This is because the reflection can

lead to the transformation between right-handed and left-handed circular polarizations. This is in contrast to the linear polarization case, where only a extra phase is added and no transformation of base vectors happens. Thus, the optical element group to perform Bell measurement consists of a BS and a HWP in such a case as shown in Figure S1. We can also easily verify its function using the way shown in text. After balancing other phase factor in the experiment, the process is described in the following:

$$\vec{E}_a, \vec{E}'_c \xrightarrow{BS} \frac{1}{\sqrt{2}}\left(\vec{E}_a - \vec{E}'_c\right) \xrightarrow{0^\circ\ HWP} \vec{E}'_{ac} = \frac{1}{2\sqrt{2}}\left(E_1 + iE_1\right)\left(\hat{h} + i\hat{v}\right). \tag{S19}$$

For such a case, $E'_{ac} = \left(E_1 + iE_1\right)/\sqrt{2}$ will be the teleported information.

After Bob receiving the teleported information that Alice sent, he will perform the correlation measurement of the first-order field by using such an information and $\vec{E}''_b = -i\left(E_r\hat{l} - E_l\hat{r}\right) = -\left(E_1\hat{h} + E_2\hat{v}\right)$. Such a process can be realized by the optical element group as shown in Figure S1. Similar to the case of the linear polarization, Bob need do orthogonal decomposition for the circular polarization field. The transmission and reflection components can be expressed as

$$\begin{aligned}\vec{E}'_{//} &= \left[E_1\cos\left(\phi + \pi/4\right) + iE_2\sin\left(\phi + \pi/4\right)\right] \\ &\times\left[\sin\left(\phi + \pi/4\right)\hat{h} + i\cos\left(\phi + \pi/4\right)\hat{v}\right]\end{aligned},$$

$$\begin{aligned}\vec{E}'_{\perp} &= -\left[E_1\sin\left(\phi + \pi/4\right) - iE_2\sin\left(\phi + \pi/4\right)\right] \\ &\times\left[\cos\left(\phi + \pi/4\right)\hat{h} - i\sin\left(\phi + \pi/4\right)\hat{v}\right]\end{aligned}. \tag{S20}$$

Here ϕ represents the rotation angle of base vectors for the circular polarization field. From the trigonometric function transformation，Eq.(S20) can be expressed as

$$\vec{E}'_{//} = -\left(\sin\phi E_r - \cos\phi E_l\right)\left(\cos\phi\hat{r} + \sin\phi\hat{l}\right)$$
$$\vec{E}'_{\perp} = -\left(\cos\phi E_r + \sin\phi E_l\right)\left(-\sin\phi\hat{r} + \cos\phi\hat{l}\right) . \quad \text{(S21)}$$

Eq.(S21) is identical with the case of the linear polarization. As $\phi = 0$, $\vec{E}'_{//}$ is the right-handed polarized light, which is identical with the polarization state of the initial incident field $\vec{E}'_c$. Similar to the case of the linear polarization, we can also obtain the following relations:

$$\left|\langle E'_{ac} E'_{//}\rangle\right|^2 = \cos^2\phi , \quad \left|\langle E'_{ac} E'_{\perp}\rangle\right|^2 = \sin^2\phi . \quad \text{(S22)}$$

This means that the angle ϕ, which is determined by the maximum of the first-order correlation between E'_{ac} and $E'_{//}$, corresponds to the teleported information. The circle dots in Figure S2 are experimental results from the correlation measurement of the first-order field for such a case. Figure S2(a) and S2(b) correspond to the results for $E'_{//}$ and $E'_{\perp}$, respectively. We can see that the maximum of the first-order correlation appears at $\phi = 0$ in Figure S2(a) and the minimum of the first-order correlation is found at the orthogonal direction as shown in Figure S2(b), which the phenomenon is similar to the case of the linear polarization.

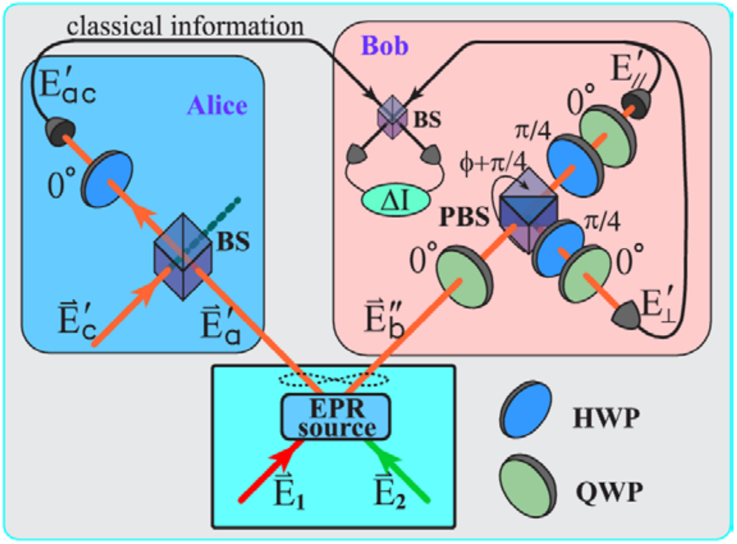

Fig. S1. Teleportation scheme showing principles and experimental set-up for the circular polarization case. The classical EPR source shown in the bottom plate. Alice and Bob share an ancillary classical entangled states marked by $\vec{E}_a$ and $\vec{E}''_b$. Alice performs a joint Bell-state measurement on $\vec{E}_a$ and the initial state marked by $\vec{E}'_c$. After Alice has sent the measured result E'_{ac} as classical information to Bob, Bob performs the correlation measurement of the first-order field by using $\vec{E}''_b$ and E'_{ac}. $E'_{//}$ and $E'_{\perp}$ represent the transmission and reflection parts as $\vec{E}''_b$ passes through a optical element group, ΔI is the difference of the light intensities at two export positions.

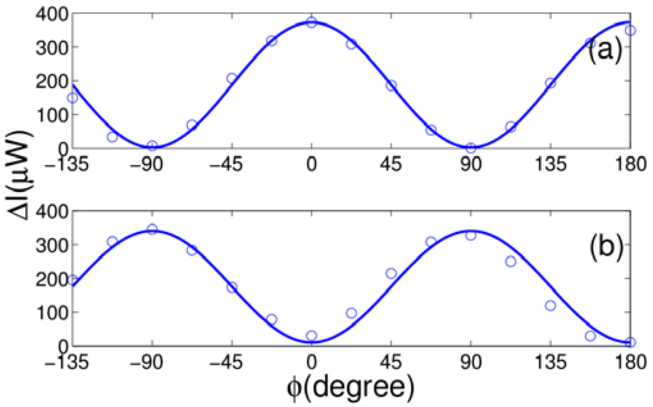

Fig. S2. Experimental (circle dots) and theoretical (solid lines) results from the correlation measurement of the first-order field for the circular polarization case, which are described by the differences of light intensities ΔI as a function of the angle ϕ. (a) and (b) correspond to the results for $E'_{//}$ and $E'_{\perp}$, respectively.
